# Leveraging Biomaterial Mechanics to Improve Pluripotent Stem Cell Applications for Tissue Engineering

**DOI:** 10.3389/fbioe.2019.00260

**Published:** 2019-10-10

**Authors:** Stephen Lenzini, Daniel Devine, Jae-Won Shin

**Affiliations:** ^1^Department of Pharmacology, University of Illinois at Chicago, Chicago, IL, United States; ^2^Department of Bioengineering, University of Illinois at Chicago, Chicago, IL, United States

**Keywords:** biomaterial mechanics, induced pluripotent stem cells (iPS cells), mechanobiology of stem cells, mechanotransduction, extracellular matrix (ECM)

## Abstract

A primary goal in tissue engineering is to develop functional tissues by recapitulating salient features of complex biological systems that exhibit a diverse range of physical forces. Induced pluripotent stem cells (iPSCs) are promising autologous cell sources to execute these developmental programs and their functions; however, cells require an extracellular environment where they will sense and respond to mechanical forces. Thus, understanding the biophysical relationships between stem cells and their extracellular environments will improve the ability to design complex biological systems through tissue engineering. This article first describes how the mechanical properties of the environment are important determinants of developmental processes, and then further details how biomaterials can be designed to precisely control the mechanics of cell-matrix interactions in order to study and define their reprogramming, self-renewal, differentiation, and morphogenesis. Finally, a perspective is presented on how insights from the mechanics of cell-matrix interactions can be leveraged to control pluripotent stem cells for tissue engineering applications.

## Introduction

**Figure 1:**
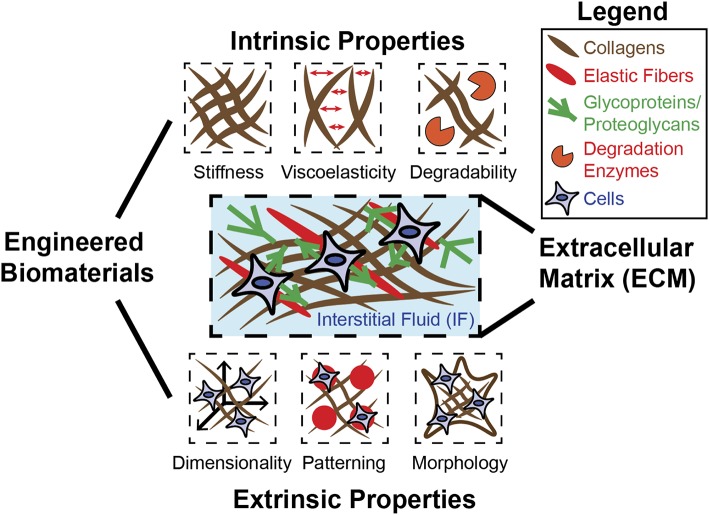
Engineered biomaterials can be used to model diverse mechanical properties of ECM. The native ECM is composed of fibrous materials (collagen, elastic fibers) as well as glycoproteins and proteoglycans, which confer adhesion between cells and the ECM. The ECM and the IF that exists within can possess many distinct mechanical properties, each of which can be recapitulated individually or in combination using biomaterial design. Intrinsic properties include stiffness, viscoelasticity, and degradability, and are generally independent of scale. Extrinsic properties include dimensionality, patterning, and morphology/geometry, and are determined by scale.

Complex biological structures arise from developmental processes, such as morphogenesis, which involves the precise transformation of materials with diverse physical properties. These materials consist of extracellular matrix (ECM), interstitial fluids (IFs) within the ECM, and cells (Forgacs and Newman, 2005). ECMs consist of fibrous components such as collagens and elastic fibers as well as glycoproteins and proteoglycans. The IF consists mainly of water and exists throughout the ECM, where it holds and transports biochemical factors critical for tissue homeostasis. The ECM and the IF jointly exhibit a diverse range of mechanical properties throughout biological systems, and the processes occurring within these systems are intrinsically tied to these properties. Embryonic development occurs within a matrix (Rozario and Desimone, 2010), and the embryo must sustain and express a diverse range of forces in order to develop into a precise and complex form. In this way, cell differentiation is fundamentally tied to force recognition and force generation (Davidson, 2017). Evidence exists for an epigenetic feedback system that allows switching of gene expression within cells depending on their morphogenic requirements (Table 1)—a system likely to be influenced by the physical environment (Smith S. J. et al., 2018). A specific example is observed in frog embryos, where mechanical properties of tissue and their relationship to the surrounding environment depend precisely on developmental stage and localization within the embryo (Shawky et al., 2018). Given these distinctions and the fact that cells exist within a diverse biophysical environment, it becomes clear that physical relationships between cell and environment are profoundly important in directing cell behaviors, including cell lineage specifications. In studies primarily involving adult stem cells, such considerations have been implemented (Shin and Mooney, 2016; Hiew et al., 2018). However, embryonic stem cells (ESCs) and morphogenesis remain to be understood with a deeper understanding of how cells generate forces and respond to mechanics of the ECM. Biomaterials can be designed to model the ECM by recapitulating biophysical properties—including intrinsic and extrinsic mechanical properties (Figure 1). Intrinsic properties such as stiffness, viscoelasticity, and degradability are based on the molecular properties of the ECM and are independent of scale (Reilly and Engler, 2010; Lee et al., 2016). Extrinsic properties such as dimensionality, patterning, and morphology are highly specific to scale and indeed very important when considering complex structures required in tissue engineering (Lee et al., 2016).

**Table 1:** Selected genes known to be involved in epigenetic feedback related to cell morphogenic requirements.

**Gene**	**Protein function**	**Roles in relation to morphogenesis**	**References**
DMNT family genes, e.g., *DMNT1*	DNA methylation	Astrocyte development; cell pluripotency; lung endoderm patterning	Freeman, 2010; Smith and Meissner, 2013; Liberti et al., 2019
HOX family genes, e.g., *Hoxb1*	Transcriptional activation and repression; intracellular signaling	Determinant of axial morphogenesis during embryonic development	Castelli-Gair Hombría and Lovegrove, 2003; Deschamps and van Nes, 2005; Bhatlekar et al., 2018
*Oct4*	Transcriptional activation and repression	Pluripotency; germ layer specification	Rossant and Tam, 2017; Mulas et al., 2018
*SIRT1*	Deacetylase activity; chromatin organization	Regulation of myogenesis; hematopoietic cell differentiation and development	Jing and Lin, 2015
*Snail*	Transcriptional activation and repression	Tissue morphogenesis; epithelial, mesenchymal, and endothelial specifications	Nieto, 2002; Dale et al., 2006; Smith S. J. et al., 2018
*Twist*	Transcriptional activation and repression	Tissue morphogenesis; epithelial-mesenchymal communications	Soo et al., 2002; Zuniga et al., 2002; Smith S. J. et al., 2018
*Wdr5*	DNA methylation	Vertebrate development; spatial tissue patterning; osteoblast and chondrocyte differentiation	Wysocka et al., 2005; Gori et al., 2006; Kulkarni and Khokha, 2018

Biomaterial approaches to precisely control intrinsic and extrinsic biophysical properties of the environment will not only enable basic investigations into how matrix mechanics regulate ESCs and their morphogenesis but will also enable engineering applications leveraged to direct ESCs and induced pluripotent stem cells (iPSCs) toward various cell fates and tissue types. This article aims to describe the relationship between biomaterial mechanics and stem cell functions while summarizing existing strategies for biomaterials to direct stem cell self-renewal, reprogramming, and differentiation. Then, the article presents a perspective on the future of biomaterial systems to implement biomaterial mechanics in iPSC applications for tissue engineering.

## Leveraging Biomaterial Mechanics to Control Pluripotent Stem Cells

Pluripotent stem cells (PSCs) are unique in their potential to undergo self-renewal and differentiation into many distinct types of cells. While ESCs were previously the major source of PSCs (Hou et al., 2013), acquisition of human ESCs from donors presents both logistic and ethical issues. Since the first demonstration of conversion from adult somatic cells into PSCs (induced PSCs, or iPSCs) (Takahashi et al., 2007), intense efforts have been made to leverage this readily accessible stem cell source to understand human development, model human disease, and regenerate human tissues. Numerous studies show that some soluble mediators, such as an inhibitors of transforming growth factor β (TGF-β) signaling, and animal-derived ECMs, such as Matrigel and collagen can be used to facilitate reprogramming of somatic cells into iPSCs (Ichida et al., 2009; Hou et al., 2013; Kim et al., 2013; Caiazzo et al., 2016), maintain self-renewal of ESCs (Watanabe et al., 2007), direct ESC differentiation into lineages (Buttery et al., 2001; Nava et al., 2012), and model organoid formation (Eiraku et al., 2011; Gjorevski et al., 2016). However, spatiotemporal presentation of soluble factors by controlled diffusion through tissues is often important to direct morphogenesis (Kinney and Mcdevitt, 2013; Lienemann et al., 2014; Zhang et al., 2018) and is typically not offered by traditional culture methods. Due to innate heterogeneities, animal-derived ECMs confound clinical translation and their properties are often difficult to control. We contend that designing biomaterials to physically control stem cell-matrix interactions would offer diverse platforms to direct pluripotent stem cells in terms of reprogramming, self-renewal, differentiation, and morphogenesis.

## Biomaterial Design to Physically Regulate Reprogramming

For successful reprogramming into iPSCs, transcription factors need to overcome the epigenetic barriers established by chromatin regulators in somatic cells (Liang and Zhang, 2013). While it is possible that incomplete or abnormal epigenetic reprogramming may not pose a problem for some applications of iPSCs (Maherali et al., 2007), iPSC-derived cells often present different phenotypes than ESC-derived cells. For example, iPSC-derived cardiomyocytes exhibit a slower beat frequency than ESC-derived cardiomyocytes (Zhang et al., 2009). Emerging studies show that the mechanics of culture environments will likely play important roles in epigenetic states of stem cells. Insights from stem cell mechanobiology already predict this observation, since the extent of nuclear lamin-A polymerization scales with matrix stiffness (Swift et al., 2013), and lamins are key determinants of how different chromatin domains interact with one another through epigenetic changes (Guelen et al., 2008; Zheng et al., 2018). A pioneering study shows that elongating fibroblasts on microgroove substrates increases iPSC reprogramming efficiency by activating chromatin regulators, such as histone H3 methyltransferase, which is necessary to overcome epigenetic barriers (Downing et al., 2013). A study with tumor-initiating stem-like cells shows that a soft fibrin-based matrix promotes histone H3 lysine 9 (H3K9) demethylation and Sex Determining Region Y-Box 2 (Sox2) expression (Tan et al., 2014), both of which are known determinants of iPSC reprogramming (Jaenisch and Young, 2008; Chen et al., 2013). Changes in epigenetic states induced by culture environments may be irreversible, as implied by a study showing that mesenchymal stromal cells (MSCs) cultured on rigid substrates for an extended period of time are no longer able to sense the difference between soft and stiff substrates (Yang et al., 2014). Thus, these studies suggest that biomaterial design can be used as an early intervention strategy to improve reprogramming by lifting epigenetic barriers.

## Biomaterials to Control Self-renewal of Stem Cells

Without self-renewal, the stem cell pool in a given system becomes depleted, which is exemplified clinically by conditions such as hematopoietic failure (Ficara et al., 2008; Wilson et al., 2009). Self-renewal of stem cells can be maintained at the level of both cell populations and single cells. At the population level, it is necessary to ensure that a fraction of stem cells is maintained, while the remaining fraction is used for differentiation or morphogenesis. In the absence of differentiation factors, ESCs proliferate rapidly in culture, as they have a significantly shorter G1 phase than other cell types (Becker et al., 2006; Fluckiger et al., 2006). However, the pool of ESCs often becomes depleted as they differentiate (Ruijtenberg and Van Den Heuvel, 2016). To overcome this issue endogenously, small fractions of stem cells are maintained in quiescence (G0 phase) *in vivo* (Cheung and Rando, 2013). Genetic studies have identified a number of factors from the bone marrow microenvironment that are required for a specific subpopulation of hematopoietic stem cells (HSCs) to undergo quiescence, such as angiopoietin-1 and stem cell factor and thrombopoietin (Arai et al., 2004; Yoshihara et al., 2007; Ding et al., 2012). Some of these factors have been conjugated with biomaterials to maintain stem cells *in vitro* (Mahadik et al., 2015). Indeed, some factors have been identified to maintain ESC self-renewal, such as basic fibroblast growth factor and leukemia inhibitory factor (Levenstein et al., 2006; Nicola and Babon, 2015). Thus, conjugating specific niche signals with biomaterials to control their spatiotemporal presentation will be useful to maintain self-renewal of a pluripotent stem cell subpopulation while simultaneously directing differentiation of other subpopulations. This strategy also presents opportunities to couple ligand presentation with biomaterial mechanics as demonstrated (Lee et al., 2011; Banks et al., 2014; Kowalczewski and Saul, 2018; Spicer et al., 2018). Alternatively, it is possible to load biochemical factors in materials that exhibit a controlled release property by designing hydrogels (Li and Mooney, 2016) to specifically couple with external stimuli such as temperature, light, affinity, or mechanical signals (Wang et al., 2017) that modulate the controlled release of biochemical factors. For example, heparin-binding-affinity-based delivery systems can be incorporated within hydrogels for simultaneously controlled delivery of several different growth factors to drive differentiation of ESCs into neural progenitors (Willerth et al., 2008). Heparin-affinity and similar systems can also be used to sequester growth factors secreted from cells (Hettiaratchi et al., 2016); for example, sequestration of growth factors secreted from co-cultured osteoblasts within heparin-containing hydrogels drives osteogenic differentiation of encapsulated MSCs (Seto et al., 2012).

At the single-cell level, self-renewal and differentiation can occur simultaneously in asymmetric cell division. During cell division, cues received through niche contact, mitotic spindle polarization, and asymmetric segregation of fate-determining molecules induce a different cell fate in a single daughter cell, while the second daughter cell remains in an undifferentiated state (Knoblich, 2008). Studies with HSCs show that asymmetric division of stem cells involves several different forces. Under external forces such as shear flow or adhesion to rigid matrices, biophysical forces become polarized toward one daughter cell, leading to asymmetric segregation of contractility molecules, such as myosin-IIB (Shin et al., 2014) and cell division cycle 42 (cdc42) (Florian et al., 2012); the daughter cell that retains these molecules remains undifferentiated. Force polarization has since been reported to control ESC self-renewal and fate specification (Maître et al., 2016) and has been used to form organized germ layers from ESCs using a soft fibrin-based matrix (Poh et al., 2014). Thus, biomaterials that control polarization of biophysical forces in dividing stem cells will be useful to maintain self-renewal while directing pluripotent stem cell differentiation.

## Biomaterial Design to Physically Direct Stem Cell Fate

Tissues exhibit a variety of physical properties. For example, bones and other tissues of mesodermal origin tend to be more rigid, while those of the neuroectoderm origin are soft. Advances in biomaterial design to precisely control material mechanics have revealed fundamental insights behind how stem cells generate forces and sense biophysical properties of the ECM during differentiation. MSCs have been used as a prototypical cell type to understand the mechanics of cell-material interactions, because they elaborate diverse cytoskeletal and nucleoskeletal machinery to sense and respond to the ECM (Discher et al., 2005). Pioneering studies leveraged engineered 2D substrates, such as polydimethylsiloxane (PDMS) and polyacrylamide-based systems, to show the importance of both cell spreading area (Mcbeath et al., 2004) and matrix stiffness (Engler et al., 2006) in directing MSC differentiation. More recent studies show that tuning substrate geometry (Kilian et al., 2010), substrate topography (Abagnale et al., 2015), and viscoelasticity (Cameron et al., 2011) of 2D substrates impacts MSC differentiation. The common conclusion of these studies is that when MSCs exhibit increased spreading and higher cortical tension on a given matrix, they undergo osteogenic differentiation. In contrast, when MSCs spread less and generate less cortical tension, they undergo adipogenic differentiation. Mechanistic studies show that increased cellular tension on stiff matrix increases polymerization of lamin-A, which leads to increased nuclear rigidity and retention of mechanosensitive transcription factors, such as Yes-associated protein (YAP), within the nucleus (Dupont et al., 2011). Nuclear retention of these transcription factors drives subsequent downstream gene expression related to osteogenesis (Swift et al., 2013). These studies suggest profound importance of 2D substrate mechanics in directing stem cell fate.

In contrast to 2D substrates, cells are often mechanically confined in 3D matrix. Therefore, the ability of stem cells to remodel and navigate through confined environments becomes important in understanding and subsequently directing stem cell functions using 3D materials. An earlier study shows that MSCs encapsulated in 3D nanoporous alginate gels that present an integrin ligand (e.g., Arg-Gly-Asp, “RGD”) undergo osteogenesis at an optimal stiffness (Huebsch et al., 2010). While this study suggests the importance of 3D matrix stiffness in directing MSC differentiation, subsequent studies show that the effect is further enhanced when MSCs are allowed to spread and generate more tension, by engineering either a metalloproteinase enzyme-degradable matrix (Khetan et al., 2013) or a matrix with faster relaxation under stress (Chaudhuri et al., 2016). Mechanotransduction pathways remain to be further defined for 3D matrix mechanics, but a recent study highlights the importance of mechanosensitive ion channels in mediating MSC differentiation in stress-relaxing materials (Lee et al., 2019). Thus, mechanical confinement and intrinsic properties of 3D materials can be tuned to control stem cell fate.

Embryonic cells secrete ECM at the earliest stages of development (Rozario and Desimone, 2010), and hence will likely be sensitive to matrix mechanics. The perinuclear region surrounding the nucleus of stem cells exhibits mechanical properties linked to nuclear structure and geometry, which is conserved across multipotent and pluripotent cells and is influenced by biochemical and biophysical inputs (Lozoya et al., 2016). Nucleus size and geometry are linked to expression of nuclear lamins A/C and emerin, which regulate gene expression during differentiation, suggesting that external mechanics are used to guide differentiation of the early embryo (Smith et al., 2017). Along these lines, PSCs exhibit high nuclear deformability that is associated with cells in specific stages of embryonic development (Grespan et al., 2018). For example, cells differentiating toward early ectoderm lineages maintain a more rigid nuclear shape while cells differentiating toward an early endoderm lineage have more deformable nuclei. Recent studies have begun to leverage biomaterials to reveal fundamental insights behind how intrinsic and extrinsic material properties direct differentiation of pluripotent stem cells. ESCs generate a higher traction force on a more rigid PDMS-based micropost array (Sun et al., 2012). While matrix stiffness does not impact self-renewal of ESCs, soft matrices facilitate mesoderm induction by stabilizing cell-cell adhesion through epithelial cadherin (E-cadherin) (Przybyla et al., 2016). A recent study used micropatterning with varied substrate geometry and size to show that iPSCs exhibit higher tension and undergo more mesodermal differentiation into vascular endothelial cadherin (VE-cadherin)^+^ endothelial cells when they are present in higher local densities (Smith Q. et al., 2018). Another study shows that there exists an optimal stiffness of a polyethylene glycol (PEG)-based hydrogel where sprouting of iPSC-derived endothelial cells becomes the maximum (Zanotelli et al., 2016).

These studies are consistent with the notion that tension generated during early gastrulation events could be an important determinant of mesoderm layer formation (Hammerschmidt and Wedlich, 2008). Thus, biomaterials can be designed to provide pluripotent stem cells with essential mechanical cues to direct their differentiation.

## Biomaterial Design for Physical Regulation of Morphogenesis From Stem Cells

Formation of highly organized tissues depends not only on spatiotemporal presentation of morphogens, substances such as TGF-β and bone morphogenic protein (BMP) that guide the patterned development of tissues, but also on precise control of physical forces. Remarkably, studies in the past decade show that some stem cells can spontaneously develop into organoids when they are cultured in animal-derived matrices such as Matrigel and collagen while in the presence of a specific combination of growth factors (Clevers, 2016). In particular, culturing ESC aggregates in Matrigel leads to an optic cup-like structure where a regional variation in tissue rigidity drives invagination (Eiraku et al., 2011), and external application of a local mechanical strain facilitates this process (Okuda et al., 2018). While these studies suggest that some cells may have the autonomous ability to coordinate physical forces during morphogenesis, cells will likely need to remodel and respond to the ECM to achieve this goal. For instance, both collagen and contractile proteins are upregulated during heart development to achieve tissue stiffness for optimal beating (Majkut et al., 2013). In addition, the ECM in the basement membrane exhibits a stiffness gradient to drive tissue elongation of the follicle during oogenesis (Crest et al., 2017). Indeed, a recent study used a PEG-based hydrogel crosslinked by a thiol-Michael addition to show that the hydrogel needs to be stiff to promote initial proliferation of intestinal stem cells, but needs to become degraded later to promote organoid formation by alleviating compressive forces (Gjorevski et al., 2016). Thus, tailoring biomaterial mechanics to a specific development process will likely determine the success of recapitulating morphogenesis from pluripotent stem cells.

## TOWARD Biomaterial Control of Nuclear Mechanics for iPSC Reprogramming

Mechanical properties of the nucleus are closely linked to chromatin states (Wang P. et al., 2018; Zheng et al., 2018) as well as cellular functions such as cell trafficking (Rowat et al., 2013; Shin et al., 2013; Harada et al., 2014) and stem cell differentiation (Shin et al., 2013; Grigoryan et al., 2018). Thus, biomaterial strategies to control nuclear mechanics will significantly impact the success of iPSC reprogramming and downstream functions. The nucleus in pluripotent stem cells is generally more pliable than in differentiated cells (Pajerowski et al., 2007). When cells with more rigid nuclei are rendered deformable by knocking down lamin-A expression, they tend to be susceptible to nuclear rupture and DNA damage induced by mechanical stress, such as squeezing (Harada et al., 2014; Irianto et al., 2017), although cells appear to possess the natural machinery to repair the ruptured nuclei during this process (Denais et al., 2016; Raab et al., 2016). Nuclear deformability is associated with upregulation of H3K4 methylation by WD repeat domain 5 (WDR5) (Downing et al., 2013; Wang P. et al., 2018), which is involved in iPSC reprogramming (Ang et al., 2011). This suggests that biomaterials that enable gradual induction of nuclear deformability without sudden mechanical stress—such as soft, stress-relaxing, or degradable substrates—can potentially enhance reprogramming efficiency while maintaining cell viability.

## TOWARD Spatiotemporal Control of Biomaterial Mechanics for Tissue Engineering With iPSCs

Since optimal mechanical properties of biomaterials will depend on different cell types and how cells are eventually organized in different tissues, solutions beyond simple bulk cell-matrix interactions will likely be required to leverage biomaterial mechanics for tissue engineering with iPSCs. From a manufacturing perspective, developing methods to precisely control biomaterial mechanics in a spatiotemporal manner using a consistent base material will lead to an economical and elegant solution to fabricate autologous tissues consisting of multiple cell types from a single iPSC source. One general strategy to achieve this goal is to combine biomaterial design with instrumentation, such as by using printers. An important advantage of this strategy is that extrinsic and intrinsic material properties can be independently controlled. In particular, light-crosslinkable materials, such as hydrogels with a methacrylate group, have been used to spatially control hydrogel crosslinking at the microscale and pattern different ligands or cells within the same substrate (Chan et al., 2010; Deforest and Tirrell, 2015; Zhang et al., 2015; Ouyang et al., 2017; Rizwan et al., 2017; Wang Z. et al., 2018). A recent example uses a printing method to spatially tune mechanical properties to simultaneously achieve adipogenic and osteogenic differentiation of MSCs in different regions of the same alginate-based substrate (Freeman and Kelly, 2017). Alternatively, biomaterials can be formed by means of temperature, pH, or even magnetic fields (Kim et al., 2016). This ability adds another dimension of possibility for precise control of patterning biomaterial mechanics (Buwalda et al., 2014), by allowing the design of biomaterials with multiscale mechanical properties, such as spatiotemporal patterning of porosity or unique topographies independent of base material properties. While precise spatial control of biomaterial mechanics remains to be leveraged for tissue engineering applications using stem cells in general, temporal control of biomaterial mechanics to control stem cell differentiation has already been demonstrated by a number of studies using light-degradable hydrogels (Kloxin et al., 2009) to dynamically control stem cell fate (Yang et al., 2014; Rosales et al., 2017). Photodegradable hydrogels have also been recently implemented in the context of 3D printing (Arakawa et al., 2017), although 3D printed photodegradable hydrogels remain to be used to mechanically direct stem cell differentiation. Biomaterials with properties sensitive to temporal application of temperature, pH, and chemical or biological stimuli have been used in other contexts such as controlled release or sequestration of molecules (Buwalda et al., 2014). Enzymatic reactions have been used to degrade biomaterials over time, leading to a temporal control of matrix properties. Actuation of biomaterials by means of temperature, pH, electricity or magnetism is possible in principle and has been demonstrated to achieve low levels of temporal mechanical control, though this concept is yet to be applied in a system to direct iPSC differentiation (Leijten et al., 2017). Nevertheless, a recent study suggests that mechanical conditioning of early-stage iPSC-derived cardiomyocytes is critical to formation of mature human cardiac tissues (Ronaldson-Bouchard et al., 2018), indicating that temporal control of mechanical cues plays an important role in directing iPSCs to form functional tissues. Thus, spatiotemporal control of biomaterial mechanics presents a promising future direction to tailor iPSCs for engineering of tissues with multiple cell types and degrees of maturity.

## Conclusions

Stem cell functions are strongly influenced by mechanical cues in the environment. Thus far, biomaterials have served a crucial role in tissue engineering by offering control of the environment and its mechanics to direct stem cell functions. The future of biomaterial strategies for improving applications of iPSCs in tissue engineering remains optimistic with a high ceiling for advancement. Due to recent advances in fields such as biomaterial design and stereolithography, research efforts are establishing biomaterial systems that allow extremely precise spatiotemporal control and presentation of mechanical cues. These systems can be designed to facilitate iPSC reprogramming, self-renewal, differentiation, and morphogenesis in a stepwise manner. Ideally, such platforms will be developed to function with minimal inputs from the end user once the primary cell-material interactions are initiated. Therefore, future challenges will involve mediating developmental transitions in a growingly diverse population of cells. Advances in material science and engineering combined with an understanding of developmental biophysics and epigenetic regulation will be critical to facilitate this modern engineering effort.

## Author Contributions

SL, DD, and J-WS wrote and contributed to the work.

### Conflict of Interest

The authors declare that the research was conducted in the absence of any commercial or financial relationships that could be construed as a potential conflict of interest.
